# Sensor-based wearable system for the detection and automatic treatment of nocturnal hypoglycaemia

**DOI:** 10.1049/htl.2018.5053

**Published:** 2018-11-05

**Authors:** Somia Sahraoui, Sofiane Sahraoui, Oussama Benbousa, Ahmed-Sami Berkani, Azeddine Bilami

**Affiliations:** 1 Computer Science Department, University of Biskra, Biskra, Algeria; 2 Department of Medicine, University of Batna, Batna, Algeria; 3 Computer Science Department, University of Batna, Batna, Algeria

**Keywords:** brain, patient treatment, diseases, blood, neurophysiology, cardiology, biochemistry, sensors, sugar, biomedical equipment, glucagon, accelerated heart-rate symptom, hyperglycaemia, sensor-based solutions, patient blood, health-related risks, daily health-related risks, diabetic patients, automatic treatment, sensor-based wearable system, progressive detection algorithm, nocturnal hypoglycaemia phenomenon, functional brain failure, glucose level

## Abstract

Diabetic patients are prone to daily and severe health-related risks, namely hyper and hypoglycaemia. Hypoglycaemia phenomenon happens when the glucose level in patient's blood is lower than a well-determined sill. It may induce serious impacts, such as functional brain failure or even the death. Hypoglycaemia is especially dangerous when it occurs during the night while the patient is asleep because it becomes difficult to be detected by the patient itself or other persons around him. While all existing sensor-based solutions are detection-only driven, the proposed solution goes beyond and attempts to treat autonomously, and at low cost, the nocturnal hypoglycaemia. The presented system detects the nocturnal hypoglycaemia phenomenon based on accelerated heart-rate symptom and a progressive detection algorithm. The system treats then the detected nocturnal hypoglycaemia throughout safe and automatic injection of glucagon.

## 1 Introduction

The rate of people with diabetes is actually terrifying and increasing continuously. In 2016, the number of diabetic persons around the world exceeded 422 million [1]. In fact, diabetic patients carry daily risks mainly represented by hyper and hypoglycaemia. Hypoglycaemia phenomenon happens when the blood-glucose level falls below the normal range (<0.7 g/l). This raises many symptoms mainly, heart-rate acceleration, tiredness, unconsciousness and so on. If not urgently faced, hypoglycaemia may lead to the death or other no less important implications.

Hypoglycaemia is commonly treated by giving sweet food to the affected person. This is neither practical nor (generally) feasible with nocturnal hypoglycaemia where the patient enters into an unconsciousness state while sleeping. Moreover, we cannot rely on surrounding persons to weak up many times during the night and check whether hypoglycaemia is happening or not with the diabetic patient.

In order to automatically deal with such a crucial problem, many sensor-based systems have been proposed: HypoMon [2] a non-invasive system that detects hypoglycaemia based on skin moisture and heart-rate acceleration signs. In [3], the authors investigate the prevention of severe nocturnal hypoglycaemia through a subcutaneous-implanted glucose monitoring system. The authors in [4], propose a solution for the avoidance of nocturnal hypoglycaemia by using predictive alarming algorithms and insulin pump suspension. Systems proposed in [5, 6] are other non-invasive solutions that are intended to detect nocturnal hypoglycaemia.

All of the existing solutions in this context share a common limitation residing in the fact that they are detective-only solutions, which is unfortunately not sufficient with nocturnal hypoglycaemia that needs in addition, to be automatically treated.

In this paper, we propose a wearable non-invasive system that targets insulin-dependent diabetics and that is able of detecting efficiently and at low cost the nocturnal hypoglycaemia. Additionally and more importantly, our system is asked to mitigate automatically such a problem. To the best of our knowledge, this is the first system that deals with the automatic treatment of nocturnal hypoglycaemia.

The purpose of this paper is before all to accentuate the necessity of the automatic treatment of nocturnal hypoglycaemia. The further main objective is to present our proposal for doing so.

## 2 Scope of the proposed system

The system is made up of two main modules: detection module and automatic treatment module.

### 2.1 Detection module

**Fig. 1:**
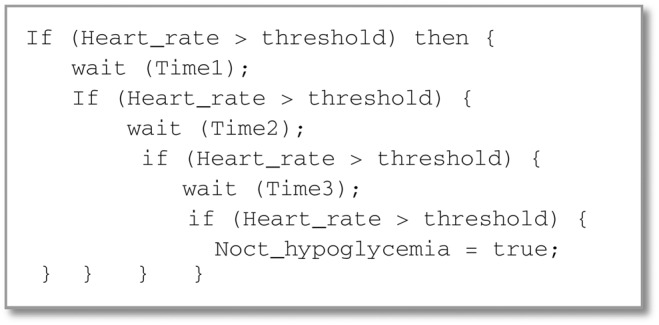
Pseudo-code of progressive hypoglycaemia detection based on heart rate sign: Time 1, Time 2 and Time 3 are fixed, respectively, to 2, 2 and 3 min. The maximal normal heart-beat threshold depends upon patient's age, e.g. with young patients (type 1 diabetes), the value is fixed to 90 beats per minute

For the detection, the system relies on the continuous heart-rate acceleration symptom, which is a strong sign of nocturnal hypoglycaemia after blood glucose deficiency. Thus, the detection module uses a heart-rate sensor for non-invasive detection that is considered as a cost-efficient solution, compared to the invasive ones. In order to ensure a good level of detection accuracy, we have introduced the algorithm depicted in Fig. 1. The algorithm shows how to make a progressive decision about hypoglycaemia occurrence in a given interval of time. Indeed, it has been experimentally determined in [7], that the continuous heart-beat acceleration during 7 min is sufficient to affirm that hypoglycaemia has happened with a diabetic patient at rest.

### 2.2 Treatment module

The system's treatment part is controlled by the detection module. Its main purpose is to deal with nocturnal hypoglycaemia upon its detection by injecting glucagon, automatically. Recalling that glucagon is the hormone responsible for getting acceptable and normal blood glucose levels. Indeed, glucagon injection can be done in a subcutaneous manner, which facilitates enough its automation. The treatment module should be composed of an injecting tool as well as, a stepper motor to launch the automatic injection and control the concentration of the glucagon to be injected. It is worth signalling at this stage that there are some special features that should be considered while manufacturing the glucagon-injecting part. In fact, the tank should carry the powder and the liquid parts of glucagon separated and mix them together after hypoglycaemia detection and just before performing the injection.

### 2.3 General functional algorithm of the proposed system

**Fig. 2:**
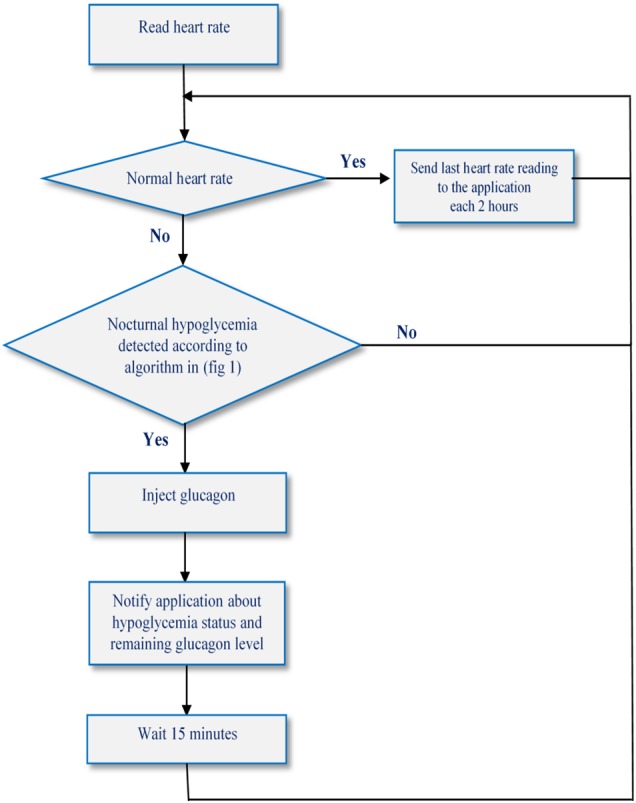
General operational algorithm of the proposed system

The whole system works according to the algorithm depicted in Fig. 2.

We suppose that glucagon tank is full before using the (wearable) system. Once the latter is operational, the sensor reads continuously the patient's heart rate and apply the algorithm presented in Fig. 1 to find out whether hypoglycaemia is happening or not. If hypoglycaemia is detected, the system activates automatically the treatment module that reacts by glucagon injection. Systematically, after injecting glucagon, the system waits for 15 min (15 min is the adequate time for the reaction of glucagon. Specialists defined it.) before performing a new test.

### 2.4 Accompanying application

**Fig. 3:**
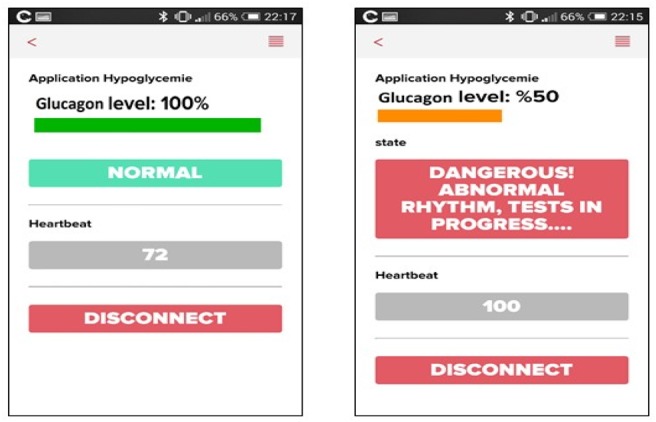
Illustration of mobile application interfaces with simulated normal and abnormal cases

Jointly, a mobile application has been developed for the notification and eventual alarming of hypoglycaemia status. The left side of Fig. 3 depicts the normal state, which means that the heart rate is normal with full glucagon tank. Such a status is reported each 2 h by the system (precisely: the sensor), as indicated in the general algorithm (Fig. 2). The right side of Fig. 3 illustrates rather hypoglycaemia status with eventually the remaining glucagon level after treatment.

**Fig. 4:**
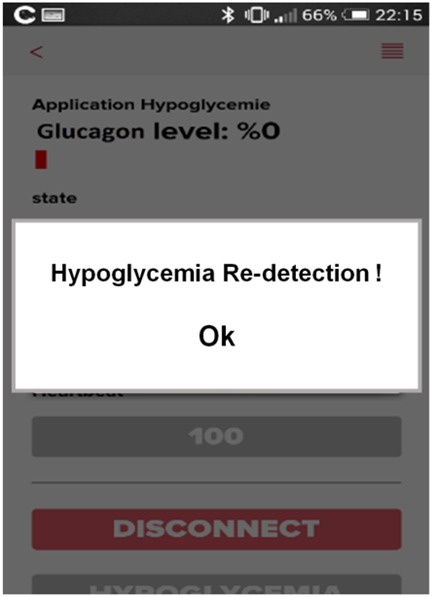
Application interface corresponding to a sonorous alarm that indicates hypoglycaemia occurrence with glucagon depletion

Additionally, the system unleashes an acoustic alarm upon hypoglycaemia re-detection with or without empty glucagon tank (Fig. 4). This allows a nearby person to wake up and help the patient to tackle the nocturnal hypoglycaemia. In the same case, and if the patient lives in an automated home, it can be involved in the system's alarming feature (e.g. upon hypoglycaemia re-detection event, the system notifies the smart home to switch on the lights).

It is worth mentioning at this stage that the proposed system does not have to alert the patient's doctor as many times as hypoglycaemia occurs. This because the doctor controls his diabetic patients systematically once a quarter.

## 3 Presentation of the realised prototype

**Fig. 5:**
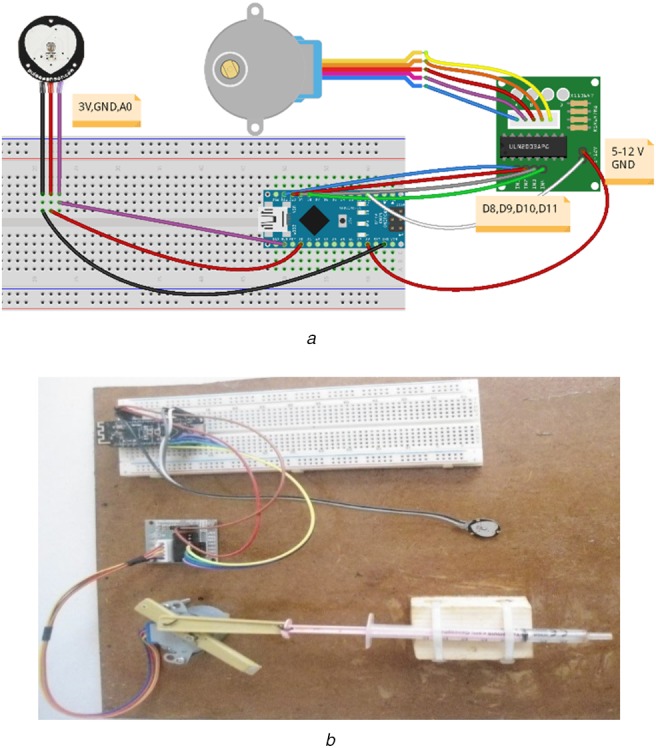
Schematisation of the prototype corresponding to the proposed system *a* Image produced by Fritzing *b* Real image

We have realised a prototype of the proposed system whose image is presented in (Fig. 5*a*) and generated with Fritzing tool [8]. The figure regroups a heart-rate sensor, a stepper 28BYJ-48 and an Arduino Nano with Bluetooth 4.0 (Bluno Nano) [9]. The real prototype image (Fig. 5*b*) includes, in addition, a syringe linked with the stepper to formally test the injection.

## 4 Performance discussion

The proposed system presents a non-invasive detection method of nocturnal hypoglycaemia based on continuous heart-rate acceleration sign and using a heart rate sensor. On the one hand, the wearable (non-invasive) healthcare systems are usually more cost-effective and flexible than the implantable ones. On the other hand, the progressive detection of hypoglycaemia that is adopted by the proposed system mitigates false-positive detection cases (e.g. the case of nightmares).

The system's detection strategy may be optionally promoted by making it smarter. This can be achieved for example by allowing the interaction between the system and other smart wearable devices, such as activity monitoring sensors so that to get useful information which will help in the (probabilistic) prediction of nocturnal hypoglycaemia. Accordingly, the necessary time for detection would not be prefixed. It could rather get reduced/extended (lower or higher than 7 min) if the system estimates an important/low probability of nocturnal hypoglycaemia.

After that, the system detects (in a non-invasive way) the nocturnal hypoglycaemia, the automatic treatment procedure that is mainly materialised by glucagon injection, should get launched. As aforementioned, hypoglycaemia causes an unconsciousness state. This way, there is no risk that the automatic injection of glucagon chocks the sleeping patient. However, appropriate needles must be used for graceful and efficient subcutaneous injection.

Before performing the injection, the system has to prepare autonomously the glucagon by mixing its powder and liquid parts together. This is a bit complicated but very approachable task that requires to be realised in a neat and flexible way.

One major advantage of the automatic treatment of hypoglycaemia by glucagon injection resides in the fact that there are no risks of hyperglycaemia nor side effects that may appear with glucagon injection. Note that in case of repeated nocturnal hypoglycaemia, the system unleashes an audible alarm that serves as a call for the traditional treatment of the problem.

Besides, the proposed system should be energy-efficient to keep operating all night long. For this reason, we suggested the use of Bluetooth 4.0, also named Bluetooth Low Energy (BLE) or smart Bluetooth [10] that is an extremely energy-effective transmission technology. The notifications that are unleashed in a regular fashion and at low-frequency (each 2 h) during the normal state, present a further efficiency factor.

## 5 Conclusion

The widespread adoption of wireless sensors in e-healthcare systems has already proven its efficiency for ubiquitous health monitoring, automatic detection or early prediction of health-related problems and many other benefits.

Regardless of their age, people with diabetes need for permanent blood glucose control in order to prevent hyper and hypoglycaemia issues. Contrary to hyperglycaemia that induces long-term dangerous impacts on the patient's body, hypoglycaemia leads to immediate and mortal consequences. Moreover, nocturnal hypoglycaemia causes frequently dead-in-bed phenomenon [11]. In this context, we proposed a seamless sensor-based system that not only detects nocturnal hypoglycaemia but treats it automatically as well.

After the realisation of a hardware and software-based testbed, along with an analytic study of the system performances, we are currently working towards the realisation of the final system that will have the form of a suited wearable bracelet.
